# Mental Health Disorders in Circumcised Reproductive-age Women, Legal Dimensions and Prevention Strategies: A Narrative Review

**DOI:** 10.1055/s-0043-1770130

**Published:** 2023-06-20

**Authors:** Farzane Alidost, Mahmoud Abbasi, Sepideh Rezaei Ghamsari, Mona Pakzad

**Affiliations:** 1 Midwifery and Reproductive Department, Tehran University of Medical Sciences, Tehran, Iran; 2 Tehran University of Medical Sciences, Tehran, Iran; 3 Shahid Beheshti University of Medical Sciences, Tehran, Iran

**Keywords:** Circumcisions, Female, Infibulation, Clitoridectomy, Genital mutilation, Mental health, Depression

## Abstract

**Objective:** Female genital mutilation/cutting (FGM/C) can affect women's lives through various physical, psychological, social and even sexual mechanisms. According to the World Health Organization guidelines for managing the health effects of FGM/C, further research into its psychological effects and preventative measures is required. In this study, a comprehensive review of the mental health consequences of circumcised women of reproductive age has been conducted with a special focus on providing preventive solutions.

**Methods:** A comprehensive search of the Web of Science, PubMed(MEDLINE), Proquest, Scopus and Google scholar was carried outfrom 2000 to 2022. The second stage of search was conducted in grey literature. To facilitate a systematic approach to search the literature, the PECO framework, was adopted.

**Results:** The result of this narrative review study showed that, the most common mental health disorder in reproductive age circumcised women were depression, anxiety and post-traumatic stress disorder. Some studies found a significant relationship between parents' education level and circumcised girls, so that parents of the circumcised women had a low level of education. Two studies considered religious beliefs, tradition, cleanness, sexual desire control and virginity as the reasons for FGM/C.

**Conclusion:** All forms of FGM/C may be harmful to one's health. Women, who have undergone widespread forms of circumcision, are more likely to develop mental disorders. As the psychosocial effects of circumcision can affect the sexual experience of circumcised women, addressing this issue, emphasizing its legal aspects, and providing preventative solutions can improve physical, mental, social, and even sexual health in circumcised women.

## Introduction

According to the World Health Organization, female genital mutilation involves partial or total removal of the external genitalia for no medical reason, which falls into four categories: Type I: the clitoris (clitoridectomy) and / or the prepuce are removed in part or completely. Type II: the clitoris and the labia minora are removed in part or completely, with or without removal of the labia majora. Type III: the vaginal orifice is narrowed with formation of a covering seal by cutting and positioning the labia minora and / or the labia majora, with or without excision of the clitoris (infibulation). Type IV: it includes all other procedures that are harmful to the female genitalia (pricking, pulling, piercing, incising, scraping, and cauterization). 1 Female genital mutilation/cutting (FGM/C) is usually performed for cultural, religious or other nonmedical reasons, 2 and it is more common in girls aged 4-10. 3 4 Although it is not clear how many women and girls undergo FGM/C worldwide, the United Nations Children's Fund estimates, that there are currently about 200 million circumcised women and girls living in 30 countries. 5 Despite the legal ban, children and women are still circumcised in 30 African countries and several countries in Asia and the Middle East. 6 The number of circumcised girls and women is increasing in Western countries due to migration. 7 Although there are no accurate statistics on FGM/C in Iran, studies show that this custom exists in some provinces, and it is common in some rural areas of southern Iran. 8

Low level of education and illiteracy, younger age, lack of knowledge about FGM/C, positive family history of FGM/C, 8 9 prevention of premarital sex, and promotion of marriage are among the common causes of FGM/C worldwide. Some communities considered female circumcision to be necessary for the transition to adulthood, and it has become a part of their cultural history and custom. 1 They also use religious interpretations to justify female circumcision, despite the fact that the Qur'an and the Bible do not support it. 10 Some ethnic groups also believe that the clitoris makes men impotent or even kills them during sex, or that the clitoris inhibits men's ability to erect. 11

Female circumcision, in addition to human rights violations, may have multiple immediate (severe bleeding, severe pain, fever, infection, shock, and even death) and long-term consequences (urinary and genital problems, sexual problems, delivery problems, reoperation and mental disorders), and it is a serious threat to their health. 1 8 Studies suggest that anxiety disorders, somatization, phobia, low self-esteem, 12 post-traumatic stress disorder, 13 affective disorders, 14 depression, 15 and memory disorders 16 are more common in circumcised women and girls.

Given the negative effects of female genital mutilation, there is now a political, national and international will to eradicate it. As the United Nations has set the eradication of FGM /C as one of its goals for sustainable development in 2030. 17 The Istanbul Convention, adopted by the Council of Europe Committee of Ministers, also recognized FGM/C as a form of gender-based violence. 18 According to the World Health Organization guidelines for managing the health effects of FGM/C, further research into its psychological effects and preventative measures is required. 19 Despite the obvious clinical and social evidence, little research into its psychological effects has been done.

The current review study aimed to examine mental health consequences of circumcision among women of reproductive age, provide preventive strategies and legal aspects of female circumcision.

## Methods

Narrative studies are considered a valuable research method in the following cases: Developing approaches to solving clinical problems, providing a voice to clients and nurses, informing social authorities and addressing diversity via understanding. 20 In addition, a narrative study is appropriate when there is limited literature for meta-analysis of the subject under study. 21 The research question in this narrative review was as follow: What are the most common psychological consequences of circumcision in women of reproductive age? Due to the relatively limited number of articles, narrative analysis was used to answer the questions.

### Search Strategy

A comprehensive search was conducted between February 1 and March 1, 2021. The search was updated in February 2022, and studies conducted on the psychological effects of circumcision on women of reproductive age were identified. Pubmed, Scopus, Proquest, Web of science, and Google scholar were searched. The grey literature was used in the second stage of the search. In this review, Population, Exposure, Comparison and Outcomes (PECO) approach has been used to develop eligibility criteria, where: Population = circumcised women of reproductive age (15-45 years); Exposure = genital circumcision; Outcome = any type of psychological or mental disorder (Stress, Depression, Post-traumatic Stress, Anxiety). We did not include comparison component in search strategy. Approach was used to generate groups of medical subject heading (MeSH) keywords. In addition, we gained access to some keywords by reviewing related articles and consulting with experts. These keywords [“psychological disorder”, “Mental disorder”, “Psychiatric illness”, “Psychiatric disease”, “Mental illness”, “Psychiatric disorder”, “Mental Health”, Stress, Depression, ”Post traumatic Stress”, Anxiety, “Genital mutilation” Clitorectomy, Infibulation, “Female genital cutting”, “Female Genital mutilation”, Circumcision] were searched and Boolean operators “OR”, “AND”, and “NOT” were used to include, restrict, and eliminate search terms. Finally, the reference list of all articles was searched for additional related studies.

### Inclusion Criteria

All studies, which were conducted on the psychological effects of circumcision on women of reproductive age from 2000 to 2022, were included without language restrictions.

### Exclusion Criteria

Studies conducted on circumcised female children or women over the age of 49 were excluded. Case reports, qualitative, methodological, mixed-method, clinical trial and review studies, studies with missing data were also excluded. By using the inclusion and exclusion criteria, 9 articles were finally included in the study and all authors agreed on the inclusion of these 9 articles.

### Selection Process

**Fig. 1:**
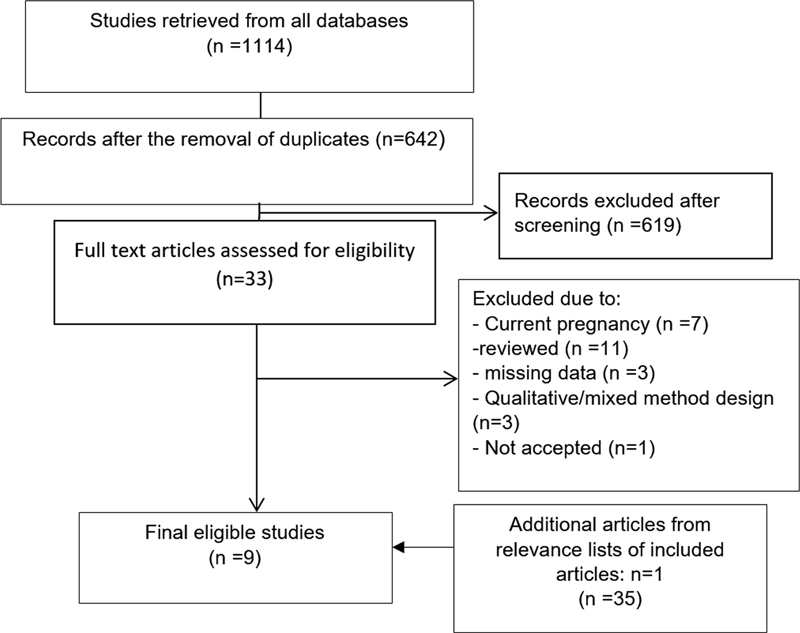
Search flow diagram.

In total, 1114 studies were extracted, which were independently evaluated by two authors (FA and MP). Duplicates were automatically removed. Then, the titles and abstracts of the remaining 642 studies were assessed and 619 more articles were excluded. Evaluating the full texts of the remaining 33 articles resulted in the exclusion of 25 ineligible articles and confirmed 9 papers as eligible ( Figure 1 ). Any cases of disagreement between authors were resolved through consensus.

### Data Extraction

Two authors (FA and MP) and a third independent reviewer (MA) reviewed the titles and abstracts of the studies. The extracted data included study characteristics (Author (year), country, Study design, Sample size, Age (year), Mean age at FGM/C, assessment tool(s), Mental health outcomes investigated, Prevalence of mental outcome (%) and a summary of relevant findings).

### Quality Assessment

The attachment of quantitative studies to the Strengthening the Reporting of Observational Studies in Epidemiology (STROBE) checklist was evaluated as a measurement tool of their quality. 22 The STROBE guidelines were created to help the author for ensuring high-quality presentation of the conducted observational study. 23 Studies were classified as high, medium, and low quality if they adhered to all seven items, six items, and two or more items of the STROBE, respectively.

## Results

### Study Characteristics

Chart 1 shows the characteristics of the included studies. All studies reviewed were observational and used a survey methodology. There were seven cross-sectional studies 3 24 25 26 27 28 29 and two case-control studies. 30 31

**Chart 1:** Summary of 9 studies evaluating the effects of female circumcision on mental health

Author (year)	country	Study design	Sample size	Age (Mean)	Mean age at FGM/C	assessment tool (s)	Mental health outcomes investigated	Prevalence (%)	Main Findings	STROBE
Behrendt e Moritz (2005) 3	Senegal	Cross-sectional	N = 47 23 circumcised women 24 uncircumcised women	22.9 ± 4.2	8.2 ± 2.7	- The Traumatic Life Event Questionnaire -semi structured interview	- PTSD - Anxiety - Affective disorders	- PTSD (30.4) - Anxiety disorders (26.2) - Affective disorders (21.7)	The circumcised women showed a significantly higher prevalence of PTSD (30.4%) and other psychiatric syndromes (47.9%) than the uncircumcised women.	16
Im et al. (2020) 24	Kenya	cross-sectional	N = 143 circumcised women	20.52 ± 3.5 (circumcised-women) 20.20 ± 3.1 (Un-circumcised women)	NR	1- PCL-C 2- HSCL-25	- PTSD - Depression - anxiety - suicidal thoughts	PTSD (38.4) - Depression (37.76) - Anxiety (38.46)	- The FGM group had much higher PTSD scores (p < .01 (, more anxiety (p < .01) and depression (p < .001), lower subjective physical health (p < .001), more trouble socializing (p < .05), more suicidal thoughts (p < .05), and greater likelihood of using substances to cope with traumas (p < .01). - Most demographic factors were not significantly associated with the FGM practice, such as age, education, country of birth, and experience of living in a camp.	20
Obaid et al. (2019) 25	Egypt	Cross-sectional	N = 200 100 circumcised women 100 uncircumcised women	22.5	NR	- HAM-A - Beck's Depression Inventory - DTS	- Anxiety - Depression - PTSD	PTSD (19%)	- No statistically significant difference between the FGM/c and control group in terms of anxiety (p = 0.37) and depression (p = 0.71).	18
Biglu et al. (2017) 26	Iran	cross-sectional	N = 208 104 circumcised-women 104 unncircumcised	27.9 ± 5.61 (circumcised-women) 27.1 ± 4.26 (Unn-circumcised women)	NR	- GHQ-28	- Insomnia - Anxiety - Severe depression - Social dysfunctions	NR	- The non-circumcised women were in better status than circumcised women regarding to the mental wellbeing (p = 0.01) - There was a significant association between FGM/C and education. the more education level of parents, the less their intensity to take their children towards FGM (p= 0.03). - Main reasons for FGM: Religious-reason (42.3%), tradition (26%), Cleanness (17.3%), Sexual desire control (9.6%) and Virginity (4.8%).	17
Daneshkhah et al. (2016) 27	Iran	cross-sectional	N = 200 140 circumcised women 60 uncircumcised women	Range: 15 - 49	NR	- GHQ-28	- Somatic symptoms - Anxiety and insomnia - Social dysfunction - Severe depression	NR	- The calculated scores for general health status did not reveal significant differences between the two groups of participants ( *P* = 0.93). - There was no significant difference in mental well-being score between the two groups (P= 0.41) - There was a statistically significant difference between the two groups in terms of parents' education level (p < 0.00). - The majority reason for FGM: religious beliefs and traditional rituals (57.1%).	20
Ibrahim et al. (2012) 28	Egypt	cross-sectional	N = 220 164 circumcised women 56 uncircumcised women	29.6 ± 8.5 (circumcised-women) 28.7 ± 6.9 (Un-circumcised women)	NR	- symptoms check list 90	- Depression - Somatization - Anxiety - Phobia	NR	- Circumcised women were found to have a lower level of education. - Type II circumcised women were found to have higher scores in the domains of somatization (p = 0.03), depression (p = 0.02), anxiety (p = 0.01) and phobia (p = 0.01).	18
Koolaee et al. (2012) 29	Iran	cross-sectional	N = 200 100 circumcised women 100 uncircumcised women	Range: 15 - 35	NR	- GHQ-28	- Sleep disorder - Depression	NR	- There were a significant difference between the two groups of participants in items of sleep disorder (p = 0.00) and general mental health between genital mutilated females and non-genital mutilated females.	19
Lever et al. (2019) 30	United States	Case-control	N = 13 circumcised women	34.0 ± 9.0	9.0 ± 6.1	- HSCL-25 - HTQR-IV	- Anxiety - Depression - PTSD	- Anxiety)92 ( - Depression)100 ( - PTSD (100)	- Survey of participants with the HSCL-25 instrument showed anxiety in 92% of participants and depression in 100% of participants. Examination of 7 participants with HTQR-IV instrument showed that all of them (100%) had PTSD criteria. - The most common symptoms in circumcised women: headaches, feeling lonely, crying and worrying too much about things, …	18
Piroozi et al. (2020) 31	Iran	Case-control	N = 247 122 circumcised women 125 uncircumcised women	35.7 ± 8.6 circumcised women 31.3 ± 7.2 uncircumcised women	NR	- GHQ-28	- Depression - Anxiety - Somatisation	- Depression (48.4) - Anxiety (62.3) - somatisation (54.1)	- More women with FGM presented with symptoms of a mental health disorder (P = 0.03). - The prevalence of symptoms of severe depression was significantly higher in the FGM group (P = 0.02). - A history of FGM and being in employment had a significant effect on presentation with symptoms of a mental health disorder (p < .05).	20

GHQ-28: The General Health Questionnaire; PCL-C: The PTSD Check List – Civilian Version; HSCL-25: Hopkins Symptoms Checklist-25; HTQR-IV : Harvard Trauma Questionnaire Revised-Part IV; HAM-A :Hamilton Anxiety Rating Scale; DTS : Davidson Trauma Scale.

### Setting

The studies were conducted in multiple countries. One study was conducted in Kenya. 25 Two studies were conducted in Egypt. 25 28 One study was conducted in the United States. 30 One study was conducted in Senegal 3 and four studies were conducted in Iran. 26 27 28 29 30

### Mental Health Assessment

Mental health was assessed through a variety of tools. Four studies used GHQ-28 questionnaire. 26 27 29 31 One study used PCL-C questionnaire. 24 Two studies used HSCL-25 questionnaire. 24 30 One study used Hamilton Anxiety Rating Scale HAM-A, Beck's Depression Inventory and Davidson Trauma Scale-DSM-IV. 25 One study used HTQR-IV questionnaire. 30 One study used The Traumatic Life Event Questionnaire 3 and another used symptoms check list 90 for Mental health assessment. 28

### Study Findings

Most of the studies showed a statistically significant relationship between depression, 24 28 30 31 anxiety, 3 24 28 30 PTSD, 3 24 30 somatization, phobia, 28 suicidal thoughts, 24 sleep disorder, 29 30 and female circumcision. Two studies did not find any statistically significant relationship between circumcised and uncircumcised women in terms of mental health disorders. 25 27 Some studies 26 27 found a significant relationship between parents' education level and circumcised girls, so that parents of the circumcised women had a low level of education while parents of the uncircumcised women had a high level of education. Two studies considered religious beliefs, tradition. 26 27 cleanness, sexual desire control and virginity 26 as the reasons for circumcision.

## Discussion

This narrative review was conducted to examine mental health consequences of circumcision among women of reproductive age, provide preventive strategies and legal aspects of female circumcision. This narrative review study examined a limited number of studies on the psychological effects of FGM /C, which is a risky social and cultural practice that threatens mental health of circumcised women. 32 Review of included studies have shown that depression, anxiety and PTSD are the most common mental health disorders in circumcised women of reproductive age. Our findings are consistent with results of prior reviews. For example, the systematic review by Abdalla and Galea (based on 16 studies) in 2019 33 and the smaller review by Berg et al. (included 4 studies), 12 that have reported association between FGM/C and adverse mental health.

The psychological consequences of female circumcision can be explained by the following mechanisms: a person's concern about the state of their genitals, future married life, and fear of infertility, or when circumcision was delayed until adolescence or early adulthood due to parental weakness or as a sort of punishment. 34 On the other hand, the education provided in schools and public forums about the negative effects of circumcision puts a lot of psychological pressure on circumcised individuals. 35 Some researchers also believe that cultural acceptance of circumcision can reduce its psychological burden. For instance, in a society where the female reproductive system is considered dirty or a source of enthralling temptation, circumcision can provide psychological relief for a girl, and despite the pain, she feels satisfied with being clean and marriageable like other women in the society. 36 On the other hand, some argue that even cultural embedment cannot protect against the psychological effects of female circumcision, such as PTSD and other psychiatric disorders. 3

Since circumcision compromises normal healthy female genital tissue and sexuality in women, it violates women and girls' rights to have the highest attainable standard of health. 6 WHO in collaboration with UNICEF and the United Nations Population Fund issued the first joint statement on FGM / C in 1997. 37 In addition, WHO in collaboration with key agencies of the United Nations and international organizations, published a document entitled “Global strategy to stop health care providers from performing female genital mutilation” in 2010. 17

The United Nations has also made the eradication of FGM / C one of its goals for sustainable development in 2003. 38 As a result of joint international efforts and legal frameworks in many countries, the number of women and men advocating for circumcision eradication is increasing, while its overall prevalence is declining. However, progress toward eradicating and reducing female circumcision is very slow. 39

Prohibition laws have been enacted in some parts of the world to reduce circumcision among girls and women and all professional associations worldwide oppose this practice. 40 In 2015, the law was expanded to require all physicians, teachers, social and healthcare providers in England and Wales to report all cases of female genital mutilation to the police directly. 41 Another preventative aspect is effective educational interventions. Denison et al. (2009) 42 showed that community empowerment through education and multifaceted social activities was more effective than training health personnel in reducing the prevalence of female genital mutilation. Education is a key indicator of protecting women from circumcision. Therefore, human rights agencies and policymakers must increase women's knowledge and awareness of the consequences of circumcision by providing educational opportunities for girls. 26 Also since children's socialization begins in the family, and they learn life skills, parents' level of education can play an effective role in transferring knowledge and attitudes to children through social learning. 32

Some researchers also believe that one of the most important ways to eradicate female circumcision is to develop the financial and executive capacity necessary to carry out basic programs and influence people in order to replace real values with harmful ones. 43 Mohamed et al. 44 also demonstrated the effectiveness of peer-to-peer workshops held in the UK to train local Somali women about female circumcision, its relationship with health and well-being, female circumcision laws and storytelling of circumcised women. In addition, religious leaders' involvement in understanding the need for change is one of effective measures in generating a transformation within culture. 36

Female circumcision can affect women's lives through a variety of physical, psychological, social, and even sexual mechanisms. In addition to the pain caused by anatomical distortion, psychological dimensions of circumcision, such as increased anxiety, depression, affecting female identity and relationship mechanisms, such as feelings of shame and marital dissatisfaction can all have a significant impact on women's sexual function. 45 As a result, public efforts should be made to raise awareness, educate girls, women, and men, and design preventive interventions in order to eliminate female genital mutilation as a form of violence against women and girls.

One of the study's limitations was the lack of evidence on the psychological effects of circumcision on circumcised women of reproductive age. Furthermore, in the studies, mental health variables were not measured with a single instrument, which may be one of the limitations of the current study.

## Conclusion

All forms of female circumcision may be harmful to one's health. Women who have undergone widespread forms of circumcision are more likely to develop mental disorders. As the psychosocial effects of circumcision can affect the sexual experience of circumcised women, addressing this issue, emphasizing its legal aspects, and providing preventative solutions can improve physical, mental, social, and even sexual health in circumcised women.
